# Aipl1 is required for cone photoreceptor function and survival through the stability of Pde6c and Gc3 in zebrafish

**DOI:** 10.1038/srep45962

**Published:** 2017-04-05

**Authors:** Maria Iribarne, Yuko Nishiwaki, Shohei Nakamura, Masato Araragi, Eri Oguri, Ichiro Masai

**Affiliations:** 1 Okinawa Institute of Science and Technology Graduate University, 1919-1 Tancha, Onna, Okinawa, 904-0495, Japan

## Abstract

Genetic mutations in aryl hydrocarbon receptor interacting protein-like 1 (AIPL1) cause photoreceptor degeneration associated with Leber congenital amaurosis 4 (LCA4) in human patients. Here we report retinal phenotypes of a zebrafish *aipl1* mutant, *gold rush (gosh*). In zebrafish, there are two *aipl1* genes, *aipl1a* and *aipl1b*, which are expressed mainly in rods and cones, respectively. The *gosh* mutant gene encodes cone-specific *aipl1, aipl1b*. Cone photoreceptors undergo progressive degeneration in the *gosh* mutant, indicating that *aipl1b* is required for cone survival. Furthermore, the cone-specific subunit of cGMP phosphodiesterase 6 (Pde6c) is markedly decreased in the *gosh* mutant, and the *gosh* mutation genetically interacts with zebrafish *pde6c* mutation *eclipse (els*). These data suggest that Aipl1 is required for Pde6c stability and function. In addition to Pde6c, we found that zebrafish cone-specific guanylate cyclase, zGc3, is also decreased in the *gosh* and *els* mutants. Furthermore, zGc3 knockdown embryos showed a marked reduction in Pde6c. These observations illustrate the interdependence of cGMP metabolism regulators between Aipl1, Pde6c, and Gc3 in photoreceptors.

In humans, genetic mutations of aryl hydrocarbon receptor interacting protein-like 1 (AIPL1) are linked to Leber congenital amaurosis 4 (LCA4), one of the inherited retinopathies^1^,^2^,^3^,^4^. LCA accounts for nearly 5% of all retinal dystrophies^5^. In general, patients who suffer from LCA are congenitally blind. Including *AIPL1*, 24 genes are associated with LCA (see the https://sph.uth.edu/Retnet). Typical symptoms of LCA include a progressive loss of photoreceptors, markedly reduced or no response of photoreceptors to light such as the electroretinogram (ERG) and nystagmus. Genetic mutations of the human *AIPL1* gene cause LCA, and in certain mutations result in dominant cone-rod dystrophy and juvenile retinitis pigmentosa^1^,^2^,^6^. However, mechanisms that underlie such diverse forms of retinal degeneration due to *AIPL1* mutations, are not fully understood.

Retinal phenotypes in mice with an *Aipl1* mutation were studied as an animal model for human LCA4. As in LCA4 patients, *Aipl1* null mutant mice show rapid degeneration of rod and cone photoreceptors in early postnatal stages^7^,^8^,^9^. Analyses of *Aipl1* mutant mice revealed that AIPL1 is required for maintenance of cGMP phosphodiesterase 6 (PDE6) α and β subunits (also referred to as PDE6a and PDE6b), which mediate rod-specific phototransduction. In *Aipl1* mutant mice combined with the knockdown of neural retina leucine zipper (NRL), almost all retinal photoreceptors are specified as cones^10^, which fail to mediate cone phototransduction via reduction of a cone-specific PDE6α’ (also known as PDE6c) and undergo degeneration^11^. Furthermore, *Aipl1* mutant mice expressing human *AIPL1* under the control of the rod-specific *Nrl* promoter (*Aipl1*^−/−^;Tg[*Nrl:hAIPL1*]) showed defects in cone phototransduction and maintenance^12^. Even though both *Aipl1*^−/−^*;Nrl*^−/−^ and *Aipl1*^−/−^;Tg[*Nrl:hAIPL1*] retinas provide artificially cone-dominated environments, these observations exclude the possibility that cone loss in *Aipl1* mutant mice results entirely from the loss of rods. Rather, they suggest that AIPL1 is cell-autonomously required for cone function and survival.

Further biochemical analyses revealed that, in the absence of AIPL1, rod and cone PDE6 subunits are synthesized normally, but are degraded through the ubiquitin-proteasome system^11^,^13^. AIPL1 regulates folding of rod and cone PDE6 subunits as a chaperone, and catalyzes their prenylation for membrane anchoring and assembly^13^. However, it remains to be determined whether AIPL1 is required only for PDE6, and whether retinopathies in LCA4 are due solely to PDE6 dysfunction.

Photoreceptor degeneration is associated with genetic mutations in components of the phototransduction cascade, including rhodopsin, PDE6b, guanylate cyclase activating proteins (GCAPs), and cyclic nucleotide-gated (CNG) channels. In the photoreceptor, under the dark condition, cGMP concentration is high, and high cGMP levels open CNG channels on plasma membranes to maintain a steady influx of Na^+^ and Ca^2+^ ions. In the light condition, PDE6 is activated to hydrolyze cGMP, resulting in closure of CNG channels. Arrest of cation influx hyperpolarizes photoreceptors. Since GCAPs are Ca^2+^ binding proteins and Ca^2+^ binding to their EF-hand motifs inhibits GCAP-mediated activation of guanylate cyclases (GC), decreased intracellular Ca^2+^ concentration caused by closure of CNG channels activates GC. Dysfunctions of PDE6, GCAPs, and CNG channels are expected to increase cGMP levels in photoreceptors, which could subsequently trigger photoreceptor cell death. However, it is unknown whether an elevated cGMP concentration induces photoreceptor cell death, and if so, what signaling pathway underlies this pathology.

Zebrafish are an animal model for research on human diseases. Moreover, zebrafish are ideal for studying cone photoreceptors since they have cone-rich retinas, and cone-visual acuity can be evaluated by an optokinetic response (OKR) at 5 day-post-fertilization (dpf). Previously we isolated the zebrafish *eclipse (els*) mutant, which shows no OKR, and found that the *els* gene encodes Pde6c^14^. The *els* mutant shows a progressive degeneration of cones, suggesting that dysfunction of cone Pde6 induces cone degeneration in zebrafish. Another group identified the zebrafish *gold rush (gosh*) mutant, which shows no OKR, and no optomotor response (OMR)^15^. Here, we investigated retinal phenotypes of the *gosh* mutant, in which cones undergo progressive degeneration. We found that the *gosh* gene encodes cone-specific Aipl1, suggesting that *gosh* mutant zebrafish provide a good model for human LCA4. Further genetic and biochemical analyses revealed interdependence between Aipl1, Pde6c, and Gc3, which supports cone photoreceptor function and survival in zebrafish.

## Results

### Zebrafish *gosh* mutant shows defects in cone opsin transport and structural integrity of subcellular photoreceptors organs

**Figure 1:**
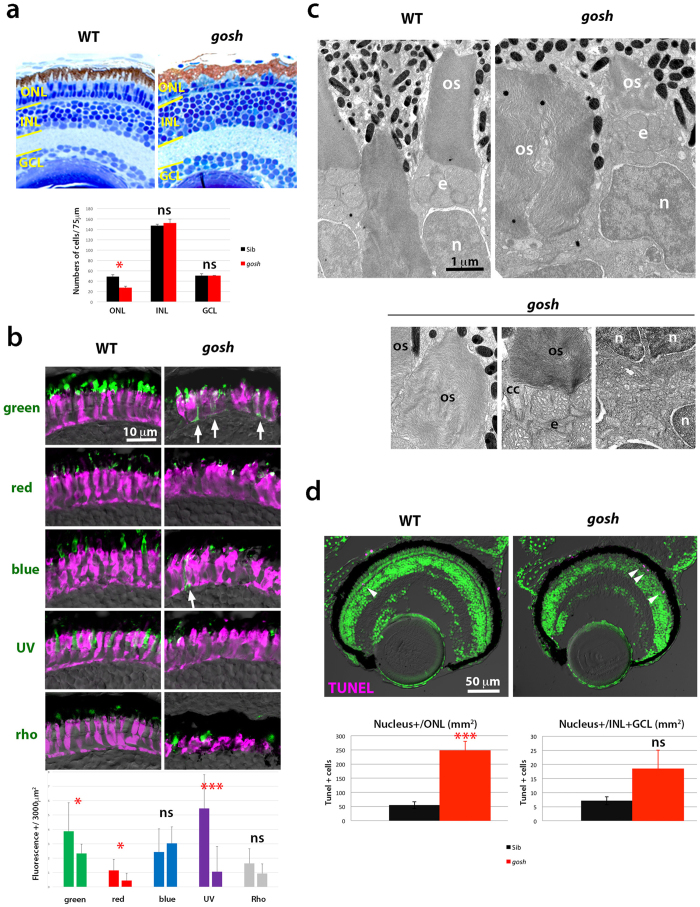
Photoreceptor phenotypes of the *gosh* mutant at 7 dpf. (**a**) (Upper) Sections of 7 dpf wild-type and *gosh* mutant retinas. In the *gosh* mutant, the ONL is thinner with abnormally shaped OS. However, INL and GCL appear to be normal. (Lower) Histogram of cell number of the ONL, INL, and GCL at 7 dpf in wild type (black) and *gosh* mutant (red). Numbers of nuclei within 75 μm length for each layer were counted (n = 3). Only ONL cells are significantly reduced in the *gosh* mutant (p = 0.026, students’ *t*-test). (**b**) (Upper) Labeling of wild-type and *gosh* mutant retinas with antibodies against green, red, blue, UV opsins, and rhodopsin (green), and zpr1 antibody (magenta). In the *gosh* mutant, the opsin-localized OS area is small, and opsins are often mislocalized to plasma membranes of cell bodies or synaptic areas (arrows). In contrast, rhodopsin localization to the OS seems to be normal. (Lower) Histogram of visual pigment-positive area at 7 dpf in wild type (left bars) and *gosh* mutant (right bars). The percentage of visual pigment-positive areas relative to a 3000 μm^2^ area containing the ONL was measured (n = 3 for each). Green, red, and UV opsin-positive areas were reduced in the *gosh* mutant, while there was no difference in blue and rhodopsin-expressing areas between *gosh* mutants and wild types. (**c**) EM images of wild-type and *gosh* mutant photoreceptors. The OS is composed of multiple, stacked photoreceptive membrane discs. Beneath the OS, mitochondria accumulate to form the ellipsoid (e). Although global shapes of the OS and nucleus (n) are deformed in *gosh* mutants, their fine structures, including the connecting cilium (cc), seem to be normal. Synaptic structures in the OPL appear to be less developed in the *gosh* mutant. (**d**) TUNEL of wild-type and *gosh* mutant retinas at 7 dpf (magenta). Apoptotic cells are indicated by arrowheads. Histogram of apoptotic cell number in the ONL (left) and the INL+GCL (right). In the *gosh* mutant, apoptotic cell number is markedly increased in the ONL. OS, outer segment; INL, inner nuclear layer; GCL, ganglion cell layer; n, nucleus; cc, connecting cilium; e, ellipsoid. (ns, p>0.05; *p<0.05; ***p<0.001).

*gosh* was identified as a mutant that shows no visual behavior due to photoreceptor depletion^15^. However, retinal phenotypes of *gosh* mutant were not investigated. Here, we examined retinal phenotypes of the *gosh* mutant histologically. Compared with wild-type retinas, the outer nuclear layer (ONL) was thinner; photoreceptors lost their columnar shape (becoming round), and the outer segment (OS) was abnormally shorter at 7 dpf (Fig. 1a). We examined cell number of the ONL, inner nuclear layer (INL) and retinal ganglion cell layer (GCL) within 75 μm along the line parallel to each layer in wild-type and *gosh* mutant central retinas (Fig. 1a). Only, ONL cells were significantly less numerous in *gosh* mutants, suggesting photoreceptor-specific defects.

Next, to examine whether all classes of cones and rods are maintained in the *gosh* mutant, we examined expression of cone opsins (green-, red-, blue- and UV-sensitive opsins) and rhodopsin. In wild type at 7 dpf, cone opsins and rhodopsin are expressed and localized to the OS (Fig. 1b). In the *gosh* mutant, opsin and rhodopsin expression was detected in the OS. However, the opsin-positive area was reduced for green, red and UV opsins, while there was no difference in the areas expressing blue opsin and rhodopsin (Fig. 1b). Furthermore, opsin expression was often not restricted to the OS, but abnormally spread to the basal region (arrows in Fig. 1b). Thus, transport of cone opsins to the OS is compromised in the *gosh* mutant.

Next, we analyzed the ultrastructure of 7 dpf retinas using electron microscopy. In wild-type photoreceptors, subcellular organs such as the OS, the mitochondria-rich ellipsoid, the nucleus, and the synapse were established (Fig. 1c). In the *gosh* mutant, these subcellular structures were normally formed, but individual photoreceptors varied in size, indicating imbalanced proportions of subcellular organs. We observed pyknotic nuclei in the *gosh* mutant ONL (data not shown), suggesting photoreceptor degeneration.

Next, we examined apoptosis using terminal deoxynucleotidyl transferase dUTP nick-end labeling (TUNEL). Apoptotic signals were mostly detected in the ONL, and increased in the *gosh* mutant (p = 0.00052) at 7 dpf (Fig. 1d). Thus, photoreceptors primarily undergo apoptosis in the *gosh* mutant.

### *gosh* mutant shows cone-specific degeneration

To examine how embryonic photoreceptor cell death influences later retinal development, we examined *gosh* mutant retinas at 4 and 12 weeks-post-fertilization (wpf). At 4 wpf we examined expression of green opsin and rhodopsin, counterstaining with zpr1 antibody, which labels double cones. The ONL was markedly thinner in the central retinas of the *gosh* mutant than in wild type retinas (Fig. S1a). Zpr1 signals were detected, but green opsin expression was almost absent, while rhodopsin expression was reduced but still observed in the *gosh* mutant (Fig. S1a). In the ciliary marginal zone (CMZ) of wild-type retinas, retinal stem cells generated all retinal cell-types, including rods and cones (Fig. S1a’). In *gosh* mutant CMZs, both cones and rods formed, and showed relatively normal columnar shapes (Fig. S1a’). However, green opsin expression was mislocalized in *gosh* mutant peripheral retinas (Fig. S1a’), while rhodopsin expression was maintained. Thus, expression of visual pigments is more severely compromised in cones than in rods in the *gosh* mutant.

**Figure 2:**
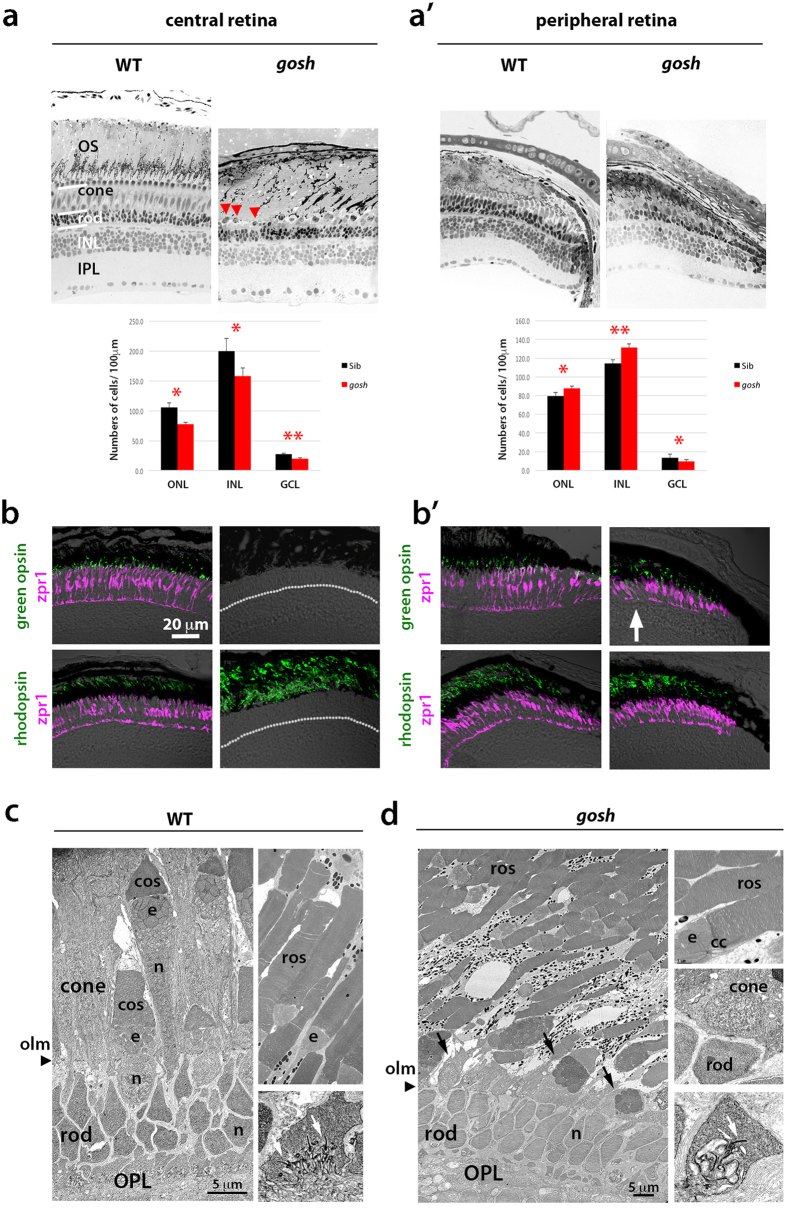
Cones are progressively eliminated in *gosh* mutants at 12 wpf. (**a**) (Upper) Sections of 12 wpf wild-type and *gosh* mutant retinas. In wild-type central retinas, rods and cones are distinguished. In *gosh* mutant central retinas, most ONL nuclei have a rod-like shapes, except for a single row of short single cones (red arrowheads), and an enlarged OS area. (**a’**) In the *gosh* mutant CMZ, both rods and cones are present. (Lower) Cell number of the ONL, INL, and GCL at 12 wpf in wild type (black) and the *gosh* mutant (red). Numbers of nuclei within 100 μm length for each layer were counted (n = 3). The ONL, INL, and GCL cells were reduced in the central retina of the *gosh* mutant. In the peripheral retina, ONL and INL cells was increased, but GCL cells were decreased in the *gosh* mutant. Students’ t-test: *p < 0.05; **p < 0.01. (**b**) Labeling of 12 wpf wild-type and *gosh* mutant retinas with anti-green opsin and rhodopsin antibodies (green), and zpr1 antibody (magenta). In wild-type central retinas, green opsin and rhodopsin are located in cone and rod OSs, respectively. However, in the *gosh* mutant, green opsin is absent, while most of the OS is labeled with rhodopsin. (**b’**) In the *gosh* mutant CMZ, both green opsin and rhodopsin are detected, although green opsin is mislocalized in some cells (arrows). A dotted line indicates the interface between the ONL and the OPL. (**c**) Twelve wpf wild-type ONL, which consists of small, compact rod nuclei, short and long single cones, and double cones (left), rod OS (right upper), and synapses (arrows show ribbon, right bottom). An arrowhead indicates the outer limiting membrane. (**d**) Twelve wpf *gosh* mutant ONL, which consists of rod nuclei and single row of cone-like cells (arrows, left), rod OS (right upper), and synapses (a white arrow shows the synaptic ribbon, right bottom). The outer limiting membrane is maintained at the outer border of rod nuclei (arrowhead). OS, the outer segment; INL, inner nuclear layer; IPL, inner plexiform layer; OPL, outer plexiform layer; olm, outer limiting membrane; ros, rod outer segment; cos, cone outer segment; e, ellipsoid; cc, connecting cilium.

Next, we examined retinal phenotypes at 12 wpf. Semi-thin sections of wild-type retinas enabled us to distinguish rods and cones according to their nuclear position and shape. Rod nuclei had small, round shapes, and formed a 3–4 cell layer above the outer plexiform layer (OPL) (“rod” in Fig. 2a). The cone layer consists of four types of cones in zebrafish, and is located above the rod nuclear layer (“cone” in Fig. 2a). Rods are elongated through the cone layer and their OS regions are located in the outer-most retinal region that associates with pigmented epithelium (“OS” in Fig. 2a). In the *gosh* mutant, almost all nuclei of the ONL showed rod-like shapes, and formed a 3-4-cell layer in the central retina. Cone-like photoreceptors were observed as a single row (Fig. 2a). In *gosh* mutant CMZs, both rods and cones were generated. We examined the number of ONL, INL, and GCL cells within 100 μm along the line parallel to each layer. Compared with wild type, cell numbers were decreased in all layers in the central retina of the *gosh* mutant (Fig. 2a). In the *gosh* peripheral retina, ONL and INL cells were more numerous, while GCL cells were less so (Fig. 2a’). Interestingly, number of rods in *gosh* mutant central retinas was 74.3 ± 3.21/100 μm (n = 3), similar to wild type (68.0 ± 2.0/100 μm, n = 3, p = 0.078), suggesting that most of the ONL cells lost in the *gosh* mutant were cones, and that rod cell number recovers by 12 wpf.

Next, to confirm cone-specific degeneration in the *gosh* mutant, we examined expression of green opsin and rhodopsin, counterstaining with the zpr1 antibody. In *gosh* mutant CMZs, both green opsin and rhodopsin expression were observed (Fig. 2b’). However, in mutant central retinas, green opsin expression and zpr1-signals were completely absent, but most of the OS area was labeled with rhodopsin antibody (Fig. 2b). Thus, cones progressively degenerate, but rods are retained to occupy the ONL in the *gosh* mutant.

We further examined ultrastructure of 12 wpf *gosh* mutant retinas using electron microscopy. In wild-type retinas, the ONL consists of rod nuclei, short single cones, long single cones, double cones and rod OS along the inner-outer axis (Fig. 2c). In the *gosh* mutant, the ONL consisted of rod nuclei and rod OS; cones were mostly absent, except for a discontinuous single row of cone-like cells (Fig. 2d). Thus, most photoreceptors in 12 wpf *gosh* mutant retinas are rods.

These cone-specific degeneration phenotypes are very similar to those of zebrafish *pde6c* mutants. In the *els* mutant, retinal regeneration was observed at 5 wpf^14^. In the zebrafish *pde6c* null allele, enhanced proliferation of INL cells occurs from 7 to 15 dpf^16^. We evaluated if retinal regeneration starts in the *gosh* mutant at 2 wpf, using a proliferating cell marker, proliferating cell nuclear antigen (PCNA)^16^. PCNA expression was observed in the CMZ, and also in the ONL and the INL, which correspond to rod progenitors and Müller cell-derived regenerating retinal progenitors in wild type. However, PCNA signals were reduced in the CMZ and in rod progenitor cells in *gosh* mutants (Fig. S1b). Although apoptosis is increased in the *gosh* mutant at 7 dpf, retinal regeneration is not active at 2 wpf. It is likely that rod number is recovered by retinal regeneration later than 2 wpf in the *gosh* mutant.

### *gosh* mutant gene encodes Aipl1b

Photoreceptor phenotypes in the *gosh* mutant resemble those of the zebrafish *els* mutant. However, *gosh* mutant retinal phenotypes, including OKR defects, were rescued by a complementation test between *gosh* and *els* mutants (all embryos showed normal OKR: n = 30 for progeny of a cross between a *gosh*^+/−^ male and an *els*^+/−^ female, n = 20 for progeny of the cross between a *gosh*^+/−^ female and an *els*^+/−^ male), suggesting that the *gosh* mutant gene is different from *pde6c*. To understand the molecular basis of the *gosh* mutation, we cloned the *gosh* mutant gene.

**Figure 3:**
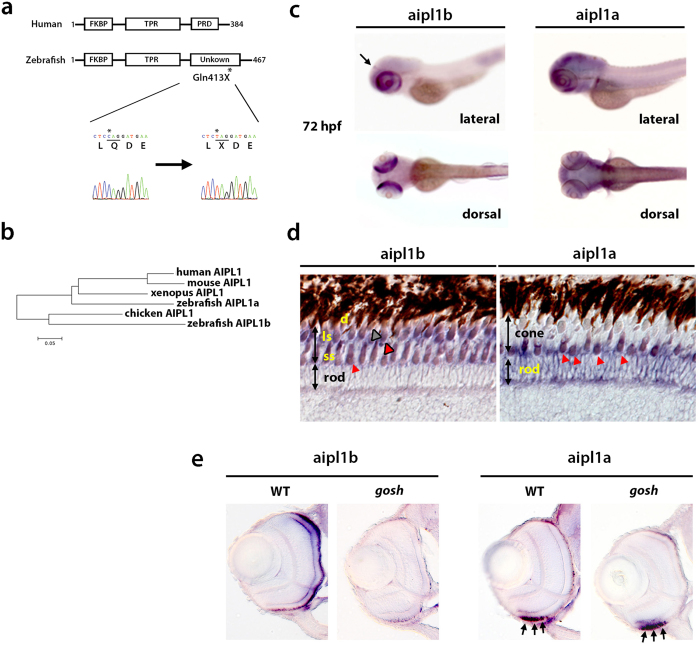
The *gosh* mutant gene encodes *aipl1b.* (**a**) Schematic representation of human AIPL1 and zebrafish Aipl1b. FKBP and TPR domains are conserved. However, the zebrafish C-terminal region is not homologous to the PRD domain. In the *gosh* mutant, a nonsense mutation occurs in Gln413. (**b**) Phylogenetic tree of AIPL1 proteins of humans (Genbank no: AAF74023.1), mice (Genbank no: AAK77956.1), chicks (Genbank no: XP_001233085.3), *Xenopus* (Genbank no: XP_002933555.1), and zebrafish Aipl1a and Aipl1b. (**c**) Expression of *aipl1a* and *aipl1b* mRNAs in wild type at 72 hpf. *aipl1b* mRNA is expressed in the retina and the pineal eye (arrow). *aipl1a* mRNA is expressed in the brain, including the retina. Strong expression of *aipl1a* mRNA in the ventral patch of the retina. (**d**) Expression of *aipl1a* and *aipl1b* mRNAs in wild-type adult retina. *aipl1b* mRNA is expressed exclusively in cones, while *aipl1a* mRNA is expressed in rods and probably in short single (UV) cones (red arrowheads). ss, short single cone (red arrowheads); ls, long single cones (red outlined arrowhead); d, double cones (gray outlined arrowhead). (**e**) Expression of *aipl1a* and *aipl1b* mRNAs in wild-type and *gosh* mutant retinas at 72 hpf. *aipl1b* mRNA is markedly decreased in *gosh* mutant retinas, while *aipl1a* mRNA expression in ventral photoreceptors (arrows) is similar between wild-type and the *gosh* mutant retina.

First, we mapped the *gosh* mutant locus onto chromosomes. Analysis using SSLP markers restricted the *gosh* mutation between 41.72 and 42.85 Mb on chromosome 14. The zebrafish genomic database indicated that 7 genes were annotated in this genomic region. Among them, we focused on a gene encoding Aryl Hydrocarbon Receptor-interacting Protein-like 1 (Aipl1), namely CT027835.1 (Clone-based Ensembl, ENSDARG00000014095.4, Ensemble Zv9 release 78), because *aipl1* is the causative gene for LCA4 in humans and is required for PDE6 function in photoreceptors. Sequencing of this *aipl1* cDNA from *gosh* mutant embryos revealed that a nonsense mutation occurred in Gln413 (Fig. 3a). Since another zebrafish *Aipl1* gene was annotated on chromosome 15 (ENSDARG00000075067.3, Ensemble Zv9 release 78), we named this gene and the *gosh* mutant gene, *Aipl1a* and *Aipl1b*, respectively. A phylogenetic tree for AIPL1 protein (Mega 6.06) revealed that zebrafish Aipl1a is more similar to human AIPL1 than to zebrafish Aipl1b (Fig. 3b). Amino acid identity between human AIPL1 and zebrafish Aipl1a was 57%, whereas 45% between human AIPL1 and zebrafish Aipl1b. Amino acid identity between zebrafish Aipl1a and Aipl1b was 39%. These data imply that the *gosh* mutant gene encodes Aipl1.

To confirm that photoreceptor phenotypes in the *gosh* mutant are caused only by the *aipl1b* mutation, we generated another *aipl1b* null allele, in which an extra 17 bp DNA fragment was inserted into the exon1 of the *aipl1b* gene (Fig. S2a), using the genome editing CRISPR/Cas9 system^17^. We confirmed that there were no OKR and photoreceptor degeneration phenotypes in the trans-heterozygotes for *gosh*^s341^ and this new *aipl1b* mutant allele *gosh*^oki6^ (Fig. S2b–c).

### *aipl1b* mRNA is expressed in cone photoreceptors

In human LCA4 patients and mouse *AIPL1* mutants, both rods and cones degenerate. In humans, AIPL1 is expressed in rods and cones during embryonic development, but is restricted to rods in the adults^18^,^19^. To determine why cones exclusively degenerate in *gosh* mutant retinas, we examined expression of zebrafish *aipl1a* and *aipl1b* genes using whole-mount *in situ* hybridization. In zebrafish, *aipl1b* mRNA was detected at the 8-cell stage, suggesting maternal expression. At 24 hpf, *aipl1b* mRNA was detected in the developing brain, but then expression began to be restricted to the retina (Fig. S3a). At 72 hpf, *aipl1b* mRNA was detected exclusively in both retinal and pineal photoreceptors, whereas *aipl1a* mRNA was expressed in the brain including the retina (Fig. 3c). Next, we examined mRNA expression of *aipl1a* and *aipl1b* in adult retina. Both genes were expressed in the ONL. *aipl1b* mRNA was expressed in all types of cones, whereas *aipl1a* mRNA was expressed in rods and probably UV cones (Fig. 3d). Double labeling of wild-type adult retinas with RNA probes for *UV opsin* and *aipl1a* or *aipl1b* confirmed that both *aipl1a* and *aipl1b* mRNAs are expressed in UV cones (Fig. S3b). Thus, the two *aipl1* genes are differentially expressed in zebrafish, and *aipl1b* mRNA is expressed exclusively in cones.

Next, we examined expression of *aipl1a* and *aipl1b* mRNAs in *gosh* mutant retinas. At 72 hpf, *aipl1b* mRNA expression was absent in *gosh* mutant photoreceptors (Fig. 3e). In contrast, *aipl1a* mRNA expression was detected in both wild-type and *gosh* mutant embryos in the ventral ONL, where rod photoreceptors start to differentiate at 72 hpf (Fig. 3e). Thus, only *aipl1b* mRNA expression is reduced in the *gosh* mutant, probably due to the nonsense mutation-mediated mRNA decay^20^. Cone-specific expression of Aipl1b explains cone opsin mislocalization and later cone degeneration in the *gosh* mutant.

### Pde6c protein is markedly decreased in the *gosh* mutant

**Figure 4:**
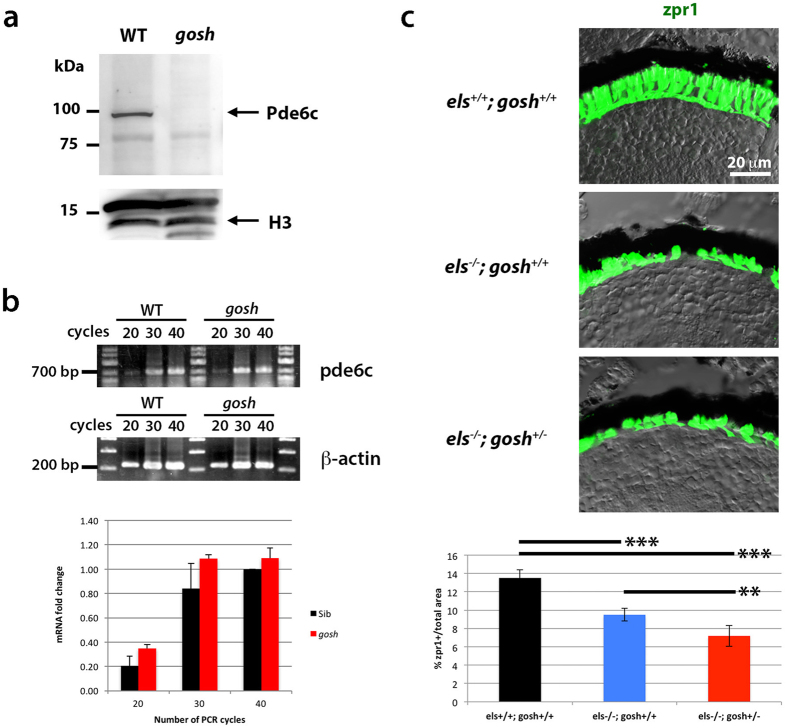
The *gosh* mutant genetically interacts with the *els* mutant. (**a**) Western blot of wild-type and *gosh* mutant heads with anti-Pde6c antibody. A band of approximately 100 kDa was detected in wild type animals, but disappeared in the *gosh* mutant. Histone H3 is as a loading control. (**b**) Semi-quantitative PCR with different numbers of cycles (20, 30 or 40) indicates that mRNA levels are similar between wild-type zebrafish and the *gosh* mutant. (**c**) Labeling of *els*^+/+^;*gosh*^+/+^, *els*^−/−^;*gosh*^+/+^, and *els*^−/−^;*gosh*^+/−^ retinas with the zpr1 antibody. The ratio of zpr1-postive area relative to the total retinal area is significantly decreased in *els*^−/−^;*gosh*^+/−^ retinas compared to *els*^−/−^;*gosh*^+/+^ retinas. ANOVA was significantly different (p< 0.000016) by t-test with a Bonferroni correction post-hoc (*p < 0.05; **p < 0.01; ***p < 0.001).

AIPL1 functions as a chaperone for rod and cone PDE6, and promotes its protein stability, its membrane anchoring via prenylation, and PDE6 subunit assembly^7^,^8^,^11^. Indeed, retinal phenotypes in the *gosh* mutant are similar to those of zebrafish *pde6c* mutants. To clarify whether *gosh* mutant phenotypes link to Pde6c dysfunction, we examined Pde6c levels. Here we used 7 dpf embryos, because at this stage, *gosh* mutants still retain photoreceptors. Western blots of wild-type zebrafish heads using anti-Pde6c antibody detected a band of ~100 kDa, corresponding to the molecular weight of Pde6c. However, in the *gosh* mutant, the band for Pde6c was undetectable (Fig. 4a). Semi-quantitative PCR indicated that the mRNA level of Pde6c did not differ between wild type and the *gosh* mutant (Fig. 4b), suggesting that protein synthesis or stability of Pde6c is compromised in the absence of Aipl1b.

### The *gosh* mutation genetically interacts with the *els* mutation

To examine whether Aipl1b directly interacts with Pde6c *in vivo*, we examined genetic interactions between *gosh* and *els* mutations. We compared photoreceptor degeneration phenotypes among 7 dpf retinas of the following three genotypes: *els*^+/+^;*gosh*^+/+^, *els*^−/−^;*gosh*^+/+^, and *els*^−/−^;*gosh*^+/−^, by immuno-labeling with zpr-1 antibody (Fig. 4c). The fraction of the zpr1-positive area relative to total retinal area in *els*^−/−^;*gosh*^+/−^ retinas was significantly smaller than that of *els*^−/−^;*gosh*^+/+^ retinas, indicating that a half dose of Aipl1b activity enhanced photoreceptor degeneration in the *els* mutant. We also confirmed that *aipl1a* mRNA level in the *gosh* or *els* mutant was similar to that of wild type, excluding the possibility that *aipl1a* mRNA expression is modified by some complementary effect or genetic feedback mechanism in both mutants (Fig. S4). These data suggest that Aipl1b directly interacts with Pde6c *in viv*o.

### cGMP concentration is not drastically increased at 4 and 7 dpf in retinal photoreceptors of *gosh* and *els* mutants

**Figure 5:**
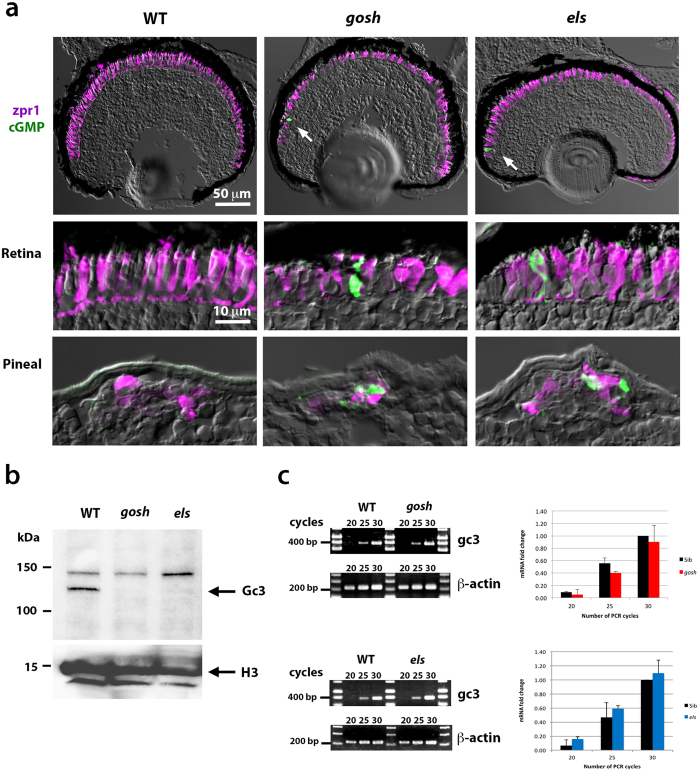
zGc3 expression is coupled to Aipl1 and Pde6c in zebrafish photoreceptors. (**a**) Labeling of wild-type, *gosh,* and *els* mutant retinas and pineal eyes at 7 dpf with anti-formaldehyde-fixed cGMP antibody (green) and zpr1 antibody (magenta). Control wild-type retinas show an undetectable level of cGMP. *gosh* and *els* mutant retinas show similar results, except that a few photoreceptors near the CMZ show very high accumulation of cGMP (arrow). On the other hand, pineal photoreceptors are positive for cGMP in *gosh* and *els* mutants, but not in wild type fish. (**b**) Western blot of wild-type, *gosh* and *els* mutant heads with anti-zGc3 antibody. A band of ~120 kDa is detected in wild-type heads, but disappears in both *gosh* and *els* mutant heads. (**c**) Semi-quantitative PCR of *gc3* mRNA expression in wild-type, and *gosh* or *els* mutant embryos. mRNA levels are similar between wild-type, and *gosh* or *els* mutant embryos.

It is likely that cGMP concentration is elevated in both *gosh* and *els* mutant photoreceptors because of severe reduction of Pde6c. It was reported that cGMP levels are elevated in a non-sense mutation allele of zebrafish *pde6c, pde6c*^w59^, at 4 dpf^21^ and in AIPL1 knockdown mice^7^. However, cGMP concentration was also reportedly decreased in AIPL1 knockdown mice^8^ and in AIPL1;NRL double mutant mice^11^. We examined cGMP concentration in wild-type, *gosh*, and *els* mutant retinas at 7 dpf, using a specific antibody against formaldehyde-fixed cGMP^22^,^23^. In light-adapted conditions, no antibody signal was observed in wild-type photoreceptors (Fig. 5a), suggesting that this antibody cannot detect background levels of cGMP. Surprisingly, we did not detect signals in either *gosh* or *els* mutant retinas in light-adapted conditions, although we sometimes observed a very strong signal in only a few photoreceptors near the CMZ (Fig. 5a). Since such positive cells overlapped with a cone marker, zpr1, these high cGMP-leveled cells are cones. In contrast to retina, we observed very strong signals in *gosh* and *els* mutant pineal glands, while no cGMP signal was observed in wild-type pineal gland. Thus, cGMP concentration is increased in pineal photoreceptors, but not in retinal photoreceptors, in both *gosh* and *els* mutants.

To investigate whether cGMP is elevated transiently during early photoreceptor differentiation but declines to low levels by 7 dpf, we labelled 4 dpf *gosh* and *els* mutant retinas with anti-cGMP and zpr1 antibodies. As at 7 dpf, we could not detect antibody signals in photoreceptors, although we occasionally observed strong signals in some photoreceptors (Fig. S5). cGMP level is not increased early in photoreceptor differentiation in either *gosh* or *els* mutants.

### Gc3 is markedly reduced in both *gosh* and *els* mutant photoreceptors

cGMP concentration is determined by the balance between PDE6 and GC activities in photoreceptors. In zebrafish, there are three retinal Gcs, zGc1–3. zGc3 is expressed in all cones, whereas zGc1–2 are expressed in rods and UV cones^24^. We examined zGc3 levels in *gosh* and *els* mutants by western blotting using a specific antibody against zGc3^25^. In wild type, the zGc3 antibody cross-reacted with a band of 120 kDa, which corresponds to the molecular weight of zGc3 (Fig. 5b). In contrast, the band was completely absent in *gosh* and *els* mutants. Semi-quantitative PCR revealed that zGc3 transcriptional level of *gosh* and *els* mutants is similar to that of wild type (Fig. 5c). Thus, Aipl1 and Pde6c are required for protein synthesis or stability of zGc3, suggesting interdependence between these cGMP metabolic enzymes in zebrafish photoreceptors.

### Pde6c is markedly reduced in the absence of Gc3

**Figure 6:**
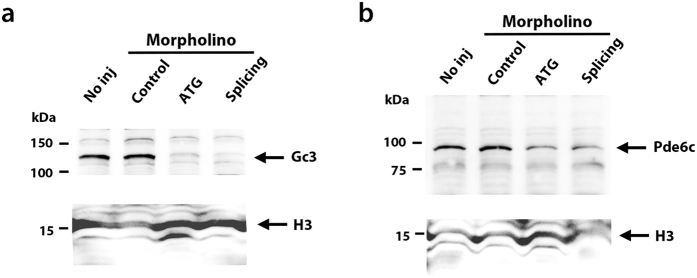
Pde6c is reduced in zGc3 morphants. (**a**) Western blot of 4 dpf wild-type and zGc3 morphant heads with anti-zGc3 antibody. A band of approximately 120 kDa was detected in wild-type and control morpholino-injected embryos, but absent in zGc3-ATG and splicing morpholino-injected embryos. Histone H3 served as loading control. (**b**) Western blot of 4 dpf wild-type and zGc3 morphant heads with anti-Pde6c antibody. A band of approximately 100 kDa was detected in wild-type, and control morpholino-injected embryos, but markedly reduced in zGc3-ATG and splicing morpholino-injected embryos. Histone H3 served as loading control.

In mice, deletion of GC1 results in no PDE6c, Transducin, and GRK1 in cone photoreceptors^26^, suggesting that GC is required for stability of phototransduction molecules. To examine whether zGc3 is required for Pde6c protein stability, we knocked-down zGc3 by injecting morpholino antisense oligos, and performed western blots of 4 dpf zebrafish heads using the anti-Pde6c antibody. First we confirmed that both ATG- and splicing morpholinos effectively inhibited zGc3 protein production (Fig. 6a). We found that Pde6c level was drastically reduced in both ATG- and splicing-zGc3 morphant embryos, whereas Pde6c was detected at normal levels in control morpholino-injected embryos (Fig. 6b). These data suggest interdependence between the regulators of cGMP metabolism, Pde6c and zGc3, in zebrafish cone photoreceptors.

## Discussion

In this study, we investigated a zebrafish *gosh* mutant that was originally identified as a visual behavior-defective mutant^15^. During embryonic stages, mutant photoreceptors show abnormal organelle shapes, defects in opsin transport, and undergo apoptosis. During adult stages, most cone photoreceptors are eliminated, but rods are retained at normal numbers and with normal structures. We found that the *gosh* mutant gene encodes cone-specific *aipl1b*, suggesting that Aipl1b is required for phototransduction and survival of cone photoreceptors.

Genetic mutations of AIPL1 cause congenital blindness, as in LCA4 in humans^1^,^2^,^3^,^4^ and rapid degeneration of rods and cones in the early postnatal period in mice^7^,^8^,^9^. Thus, photoreceptor degeneration phenotypes in the zebrafish *gosh* mutant seem to be consistent with those caused by human and mouse AIPL1 mutations. In the zebrafish *gosh* mutant, photoreceptors show abnormal proportions of subcellular structures and increased cell death at 7 dpf, suggesting a possible role of Aipl1 in photoreceptor differentiation. For the future development of therapy for human LCA4 patients, it is important to address whether photoreceptor differentiation is compromised in the absence of Aipl1 activity. The zebrafish *gosh* mutant may provide a good model for studying this issue.

Photoreceptor phenotypes in the *gosh* mutant are similar to those of zebrafish *pde6c* mutants^14^. AIPL1 promotes stability of PDE6c, and membrane anchoring via prenylation^11^. We found that Pde6c is markedly decreased or absent in *gosh* mutants. However, its mRNA level is not affected. Thus, the absence of Aipl1 compromises Pde6c protein synthesis post-transcriptionally or protein stability. Furthermore, genetic interaction between the *gosh* and *els* mutations indicates that Aipl1b directly interacts with Pde6c *in vivo*. These data support the model that AIPL1 functions as a chaperonin for PDE6. Thus, the requirement of AIPL1 in PDE6 functions is conserved from fish to humans. We have no data indicating that Aipl1 is required for rod Pde6 functions in zebrafish, because *aipl1a* mutants have not been reported. It will be interesting to examine whether rod Pde6 levels are reduced in zebrafish *aipl1a* mutants.

It was reported that genetic mutations of phototransduction molecules, including PDE6b, GCAPs, and CNG channels, cause photoreceptor degeneration. In all these cases, cGMP accumulations are expected, and these may trigger photoreceptor degeneration. However, decreased cGMP concentrations were also reported in *Aipl1* mutant mice^8^,^11^. Thus, it is important to examine cGMP levels in *gosh* mutant photoreceptors. We found that cGMP concentration is not drastically elevated in retinal photoreceptors in light-adapted *gosh* and *els* mutants at 4 and 7 dpf. This is surprising because cGMP level are reportedly elevated in the zebrafish *pde6c* null mutant, *pde6c*^w59^, at 4 dpf^21^. This discrepancy may be due to differences in the anti-cGMP antibodies used, or allelic differences between *pde6c*^w59^ and our allele, *els (pde6c*^rw76a^). In the latter case, cGMP concentration may be increased in *gosh* and *els* mutants, but less than the threshold level that the antibody can detect. However, since zGc3 is almost absent in *gosh* and *els* mutants at 7 dpf, it is likely that this reduced zGc3 level suppresses chronic elevation of cGMP. In *Aipl1;Nrl* double knockout mice, PDE6c was not maintained, but cGMP level was reduced in dark-adapted conditions^11^. In this double mutant, RetGC1 protein level is also reduced. Thus, the absence of PDE6c activity is directly linked to instability of GC proteins throughout vertebrate photoreceptors.

Both rod and cone PDE6 subunits are markedly decreased or absent in *Gc1*^−/−^;*Gc2*^−/−^ double mutant mice^26^. Thus, reduction of GC activity causes PDE6 instability in mice. We found that Pde6c is reduced in zGc3 morphants at 4 dpf, suggesting interdependence between Pde6c and zGc3. At the moment, the molecular mechanism underlying the coupling of Pde6 and Gc3 maintenance in photoreceptors is unknown. One possibility is that intracellular transport of Pde6c and Gc3 is shared in zebrafish photoreceptors. PrBP/δ, also known as PDE6d, which was originally identified as a rod PDE6 subunit, regulates transport of rhodopsin kinase (GRK1) and PDE6c to the OS^27^. Furthermore, both PDE6c and GRK1 were undetectable in the absence of GC1^26^, raising the possibility that these proteins are transported to the OS with GC1-containing transport vesicles. However, RD3, mutations of which cause LCA12 in humans, binds to GC1 and GC2, and regulates their transport to the OS in mice^28^. In RD3 knockdown mice, PDE6c levels are normal, suggesting that transport of PDE6c to the OS is independent of RD3, and probably different from that of GC1. In the latter case, coupling of PDE6c and GC3 maintenance may be mediated by a physiological condition, rather than by a direct interaction between them. It is important to address this topic in the future.

Another important question is how photoreceptor cell death is induced in the *gosh* mutant. Similar retinal phenotypes between *gosh* and *els* mutants suggest a common cell death mechanism, which is presumably linked to the absence of Pde6c. In the mouse *Pde6b* mutant, rod cell death is induced by apoptosis in a cell-autonomous manner, whereas cone cell death is induced cell-non-autonomously by necroptosis, which depends on receptor-interacting protein kinases 1 and 3 (RIP1 and RIP3)^29^. Recently, it was reported that photoreceptor cell death in the zebrafish *pde6c* null mutant, *pde6c*^w59^, depends on Rip3^21^. The authors showed that *rip*3 knockdown rescues cell death, and visual responses in zebrafish *pde6c* mutant. They speculated that rod Pde6 subunits may be artificially expressed in *pde6c* mutant cones, where they mediate cone phototransduction even in the absence of Pde6c. However, we did not observe OKR in zebrafish *gosh* or *els (pde6c*^rw76a^) mutant embryos injected with morpholino antisense of *rip3* (data not shown). Furthermore, it was reported that cone cell death in zebrafish *pde6c*^w59^ mutants is induced cell-autonomously^30^, in contrast to the cell-non-autonomous necroptotic cone cell death model^31^. Further investigation is required to understand cell death mechanisms in zebrafish *aipl1b* and *pde6c* mutants.

The secondary loss of healthy neurons surrounding dead or dying neurons harboring primary genetic defects is known as bystander effect^32^. Such a bystander effect was reported in human retinitis pigmentosa, in which rod photoreceptors firstly die, followed by later cone degeneration. However, cone-dystrophy diseases do not induce the secondary death of rods^33^. In the *gosh* mutant, cones progressively degenerate. At the embryonic stages, rods are deformed and some of them are likely to degenerate. However, at the adult stages, rod number is recovered to a normal level. In the *pde6c* null mutant, *pde6c*^w59^, rod degeneration is limited to areas with a low rod density^34^. A cone-specific knockdown of Ran-binding-protein-2 (Ranbp2) in mice induces cone degeneration, but the secondary loss of rods only occurs when degenerating cones are present^35^. Thus, the early transient rod degeneration in the *gosh* mutant may associate with the presence of degenerating cones, although its underlying mechanism is unknown.

Unlike mammals, zebrafish can regenerate a damaged retina^16^. Although photoreceptors undergo apoptosis at 7 dpf in the *gosh* mutant, retinal regeneration is not initiated at 2 wpf. This is a bit surprising, because INL cell proliferation is elevated in the null allele of zebrafish *pde6c* mutant at 7 dpf^16^. Although our western blot did not detect Pde6c protein in the *gosh* mutant, some residual Pde6c activity may delay retinal regeneration. Consistently, we observed enhanced INL cell proliferation in the *gosh* mutant retinas at 7 wpf (data not shown). Since such later regeneration can explain the early transient rod degeneration in the *gosh* mutant, it is important to investigate the interrelationship between cone degeneration and rod regeneration.

## Material and Methods

### Ethics statement

All zebrafish experiments were performed in accordance with the Animal Care and Use Program of Okinawa Institute of Science and Technology Graduate University (OIST), which is based on the Guide for the Care and Use of Laboratory Animals by the National Research Council of the National Academies and has been accredited by the Association for Assessment and Accreditation of Laboratory Animal Care (AAALAC International). All the experimental protocols were approved by the OIST Institutional Animal Care and Use Committee (Approval ID: 2014-83~86).

### Fish

Zebrafish (*Danio rerio*) were maintained according to standard procedures^36^. Okinawa wild type (*oki*) was used as a wild-type strain. WIK was used for mapping the *gosh* mutation. The *gosh* mutant was originally isolated in the screening of zebrafish visual mutants using a chemical mutagen, N-ethyl-N-nitrosourea (ENU)^15^. A zebrafish *pde6c* mutant, *els*, was identified in our previous screening of zebrafish visual mutants^14^. *gosh*^s341^, *gosh*^oki6^, and *els*^rw76a^ were used for our analyses. OKR was performed to identify visual mutants at 5–7 dpf following the published method^37^. Larvae were partially immobilized in a petri dish containing methylcellulose. A drum with black and white vertical stripes (18°) was placed around the petri dish, and turned around at 10–20 rpm.

### Mapping, and cloning of the *gosh* mutant gene

Mapping of the *gosh* mutation was conducted as described previously^38^. Two polymorphic markers, CH174K20 and zK5I9, were designed using genomic database information (Ensemble Zv9 release 78) at positions 41.72 and 42.85 Mb on chromosome 14, respectively. These markers flank the *gosh* mutation. For cloning of the *gosh* mutation, we surveyed all genes aligned between CH174K20 and zK5I9, and identified one of *aipl1* genes, namely *aipl1b*. cDNA fragments encoding zebrafish *aipl1b* were cloned from total RNA prepared from 7 dpf *gosh* mutant embryos using PCR, and sequenced using a DNA sequencer (3130xl Genetic Analyzer, Applied Biosystems).

*aipl1b* forward primer, 5′-CAAGGCTACAGGACATTAGGACTG-3′

*aipl1b* reverse primer, 5′-CCAAACGTACCTGTGCAACTTAAGAC-3′.

### Isolation of a new *aipl1b* mutant allele using the CRISPR/Cas9 genome editing system

Guide RNA (gRNA) was designed using the webpage http://crispr.mit.edu/. A 20 bp sequence targeted to exon 1 of the *aipl1b* gene, ACGTATCTGCTAAACCATCC, was selected as gRNA-1. A chimeric sgRNA, consisting of pT7-gRNA^17^ and the AIPL1b target sequence, was generated by *in vitro* transcription using a MEGAscript T7 kit (Ambion), and purified with an RNeasy kit (Qiagen). For Cas9 preparation, we used pCS-nCas9n, a PCS2 plasmid construct that expresses nls-zCas9-nls^17^. Capped mRNA encoding nls-zCas9-nls was synthesized using a mMESSAGE mMACHINE SP6 kit (Ambion). Poly(A) tailing was added with *E. coli* Poly (A) Polymerase (NEB# M0276), and then purified by column purification (QIAprep, Qiagen). A mix of 30 ng/μL of sgRNA and 75 ng/μL nls-zCas9-nls RNA was injected into one-cell**-**stage zebrafish embryos. The F1 generation was produced by a pair-wise cross of the F0-injected adult fish. F1 fish in which deletion or insertion occurred at the target region of exon1 were identified by PCR amplification of the target region. By this means, we established a fish line that has a 17 bp insertion, as a new allele of the *gosh* mutant (*gosh*^oki6^), which produces truncated Aipl1b due to an immature stop codon in exon 1.

### Morpholino antisense-mediated knockdown of zGc3

We used two morpholino antisense oligonucleotides for gc3 (GeneTools, Inc., Philomath, OR), which targeted the 5′-untranslated region (ATG), or splicing site (splicing), respectively. Standard morpholino was used as a control. Morpholino was dissolved in water at 0.25 mM and injected into wild-type embryos at the one-cell stage. MO sequences are shown below.

MO-gc3-ATG, 5′-TTGTTGAATATCAGAGACGAAGCGA-3′

MO-gc3-splicing, 5′-ATGATGACAACTGCACACAACAAAT-3′

Standard MO, 5′-CCTCTTACCTCAGTTACAATTTATA-3′.

### Histology

*In situ* hybridization, plastic sectioning, immunolabeling of cryosections and paraffin sections were performed as described previously^38^. Paraffin sections were pretreated with heat (120 °C, 20 minutes, in 10 mM citrate buffer pH 6.0). zpr1 antibody (ZIRC, Eugene, Oregon; 1:100), anti-zebrafish rhodopsin (1:5000), red opsin, green opsin, blue opsin, and UV opsin (1:200–1000)^39^, and PCNA (clone PC10, Sigma P8825; 1:200) were used. Antibody against formaldehyde-fixed cGMP^40^ (1:1000) was used for immunolabeling. TUNEL was performed using an *In Situ* Cell Death Detection Kit (Roche) and carried out as previously described^14^. Nuclear staining was performed using 50 nM SYTOX Green (Molecular Probes) or 1 nM TOPRO3 (Molecular Probes). Images were scanned using a confocal laser scanning microscope (Carl Zeiss, LSM510 and LSM710). EM analyses were performed as previously described^14^, and examined with an electron microscope (JEM 1230; Jeol Co. Ltd., Tokyo, Japan).

### Western blots

Pools of 5 heads isolated from 4 or 7 dpf embryos were used for western blotting. Protein purification and SDS-PAGE were conducted as previously described^41^. Anti-Pde6c (Abcam; ab5660), zGc3^25^, and histone H3 (Abcam; ab1791) antibodies were used at dilutions of 1:2000, 1:1000 and 1:50000, respectively. Signals were developed using horseradish peroxidase (HRP)-conjugated secondary antibodies (GE Healthcare Life Sciences, NA934VS, 1:5000) and ImmunoStar LD (Wako). Luminescence was quantified using a Fuji LAS 4000 image analyzer (Fuji Photo Film).

### Semi-quantitative PCR

Total RNA was prepared from 7 dpf *gosh* and *els* mutant embryos. Complementary DNA strands were synthesized from total RNA using an AMV reverse transcriptase and used as a template for semi-quantitative PCR. cDNAs of pde6c, zGc3, aipl1a, and β-actin were amplified with the RNA LA PCR^TM^ kit (TaKaRa Biochemicals), either with 20, 25 or 30 cycles; or 20, 30 and 40 cycles. The following primers were used. Amounts were quantified using ImageJ (NIH).

pde6c forward, 5′-CTCCACTCATGCAGGGAAAAG-3′

pde6c reverse, 5′- GTAATCTGTTCATCCTGCTCTTCG-3′

zGC3 forward, 5′-GGCACATGTTGGGATTGTAACTG-3′

zGC3 reverse, 5′-GGTTACTGTTAAGACCCCATCG-3′

aipl1a forward, 5′-GGACTGGCACATTCACACCTG-3′

aipl1a reverse, 5′-GCCACGCATATAGAAGGCTTTC-3′

β-actin forward, 5′-GAAATTGTCCGTGACATCAA-3′

β-actin reverse, 5′-GAAGGTGGTCTCGTGGATAC-3′.

## Additional Information

**How to cite this article**: Iribarne, M. *et al*. Aipl1 is required for cone photoreceptor function and survival through the stability of Pde6c and Gc3 in zebrafish. *Sci. Rep.* **7**, 45962; doi: 10.1038/srep45962 (2017).

**Publisher's note:** Springer Nature remains neutral with regard to jurisdictional claims in published maps and institutional affiliations.

## Supplementary Material

Supplementary Materials
