# Supplementary material for: Aipl1 is required for cone photoreceptor function and survival through the stability of Pde6c and Gc3 in zebrafish
Source: Sci Rep. 2017 Apr 5;7:45962. doi: 10.1038/srep45962 (PMC5381001; doi:10.1038/srep45962)
Supplement: Supplementary Materials [file srep45962-s1.pdf]

Supplementary materials

Aipl1 is required for cone photoreceptor function and survival through the stability of Pde6c and Gc3 in zebrafish

Maria Iribarne, Yuko Nishiwaki, Shohei Nakamura, Masato Araragi, Eri Oguri, and Ichiro Masai

Supplementary figures: Figure S1-5

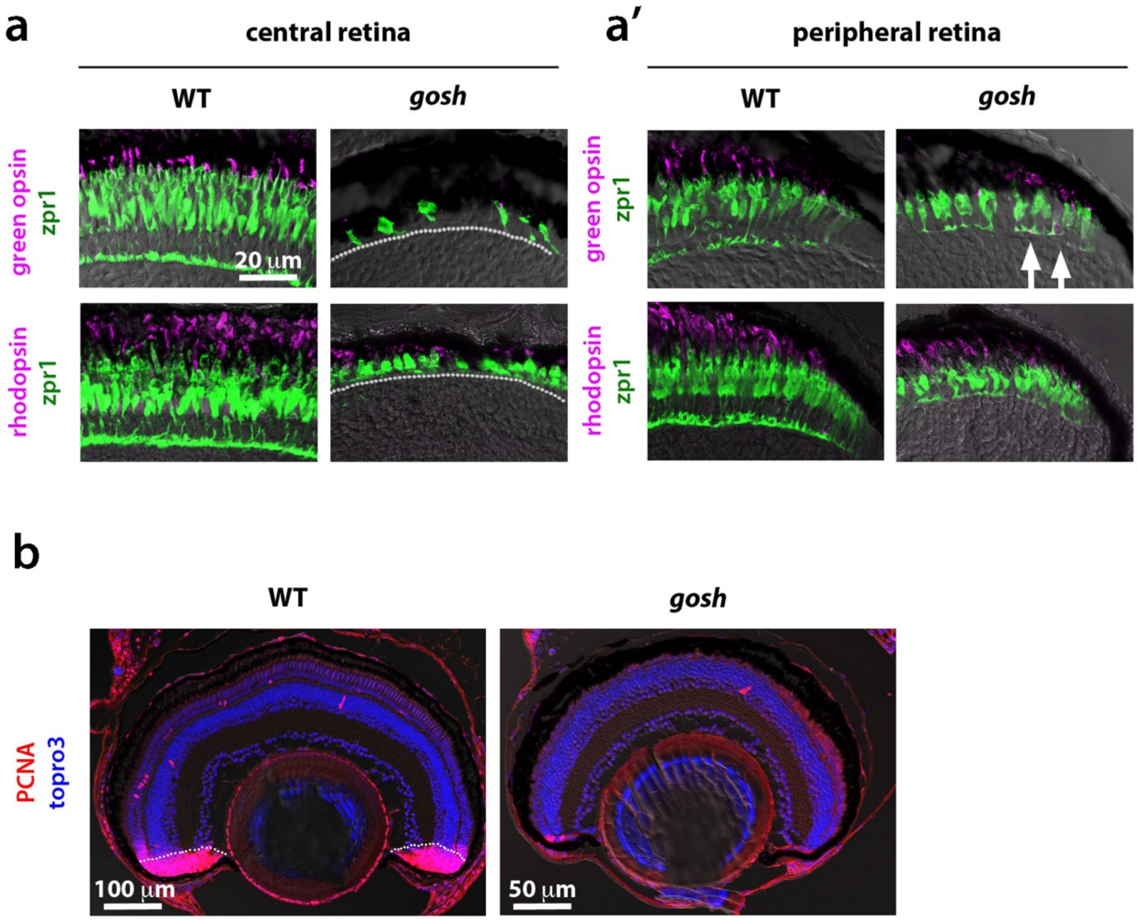

Figure S1. Degeneration of cone and rod photoreceptors at 4 wpf in the *gosh* mutant

(a) Labeling of wild-type and *gosh* mutant retinas at 4 wpf with green opsin and zpr1 antibodies, or rhodopsin and zpr1 antibodies. In wild-type central retina, the ONL is thicker than at 7 dpf to segregate rod and cone nuclear layers. Its cone shape is highly elongated. Opsin and rhodopsin are localized in the OS. In *gosh* mutant central retina, the ONL is very thin, and cones are fewer in number and abnormal in shape. (a') In wild-type peripheral retina, new rod and cone photoreceptors are generated and express rhodopsin and opsin, respectively. In *gosh* mutant peripheral retina, cones become columnar although their thickness is still less than in wild type retina. Green opsin and rhodopsin are detected; however, green opsin is mislocalized through the cell body (arrows). A dotted line indicates the boundary between photoreceptor cells and INL.

(b) Labeling of wild-type and *gosh* mutant retinas of 2 wpf with PCNA antibody (red). Nuclei are counter-stained with TOPRO3 (blue). In wild-type retina, most of the CMZ express PCNA, indicating cell proliferation of retinal stem and progenitor cells (dotted line). In wild-type retina, PCNA positive cells are observed just beneath the ONL and in the INL, which correspond to rod progenitors and Müller cell-derived regenerating progenitors, respectively. In contrast, PCNA expression is reduced in the *gosh* mutant CMZ, in the number of rod progenitors and Müller cell-derived regenerating progenitors, suggesting that retinal regeneration is not active in the *gosh* mutant at 2 wpf.

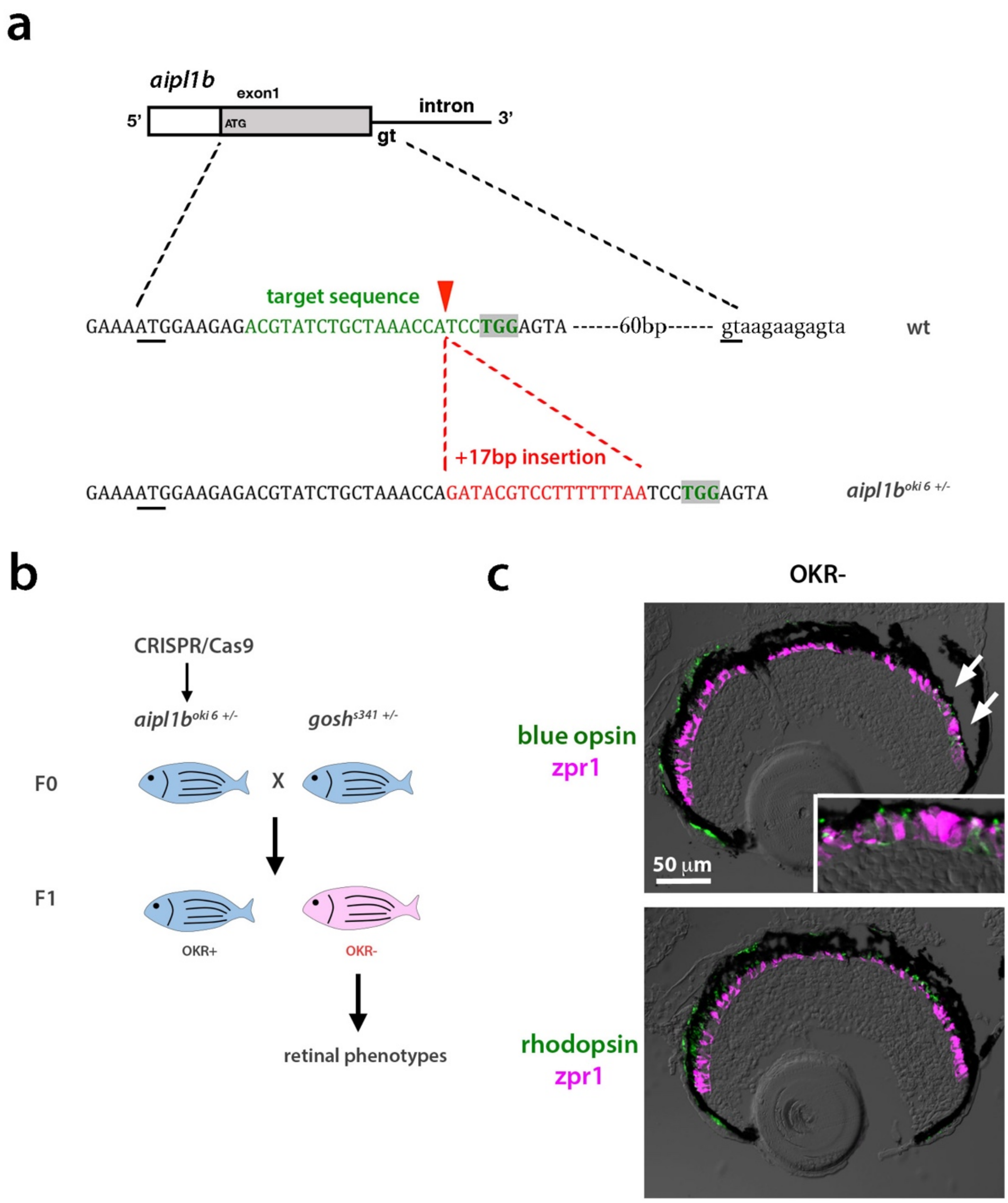

Figure S2. Generation of a new allele of the *gosh* mutant using the CRISPR/Cas9 system

(a) Sequence of exon1 of the *aipl1b* gene in embryos showing no OKR response. A 17-bp insertion causes a frame-shift of the coding region, which subsequently induces a premature stop codon.

(b) Diagram showing the strategy used to confirm that *gosh* mutant gene encodes *aipl1b*.

(c) Labeling of retinas of 7-dpf no-OKR zebrafish embryos with anti-blue opsin and zpr1 antibodies or anti-rhodopsin and zpr1 antibodies. Photoreceptors are abnormal in shape, and blue opsin is mislocalized to the plasma membrane of the cell body (arrows). However, rhodopsin is normally localized at the OS.

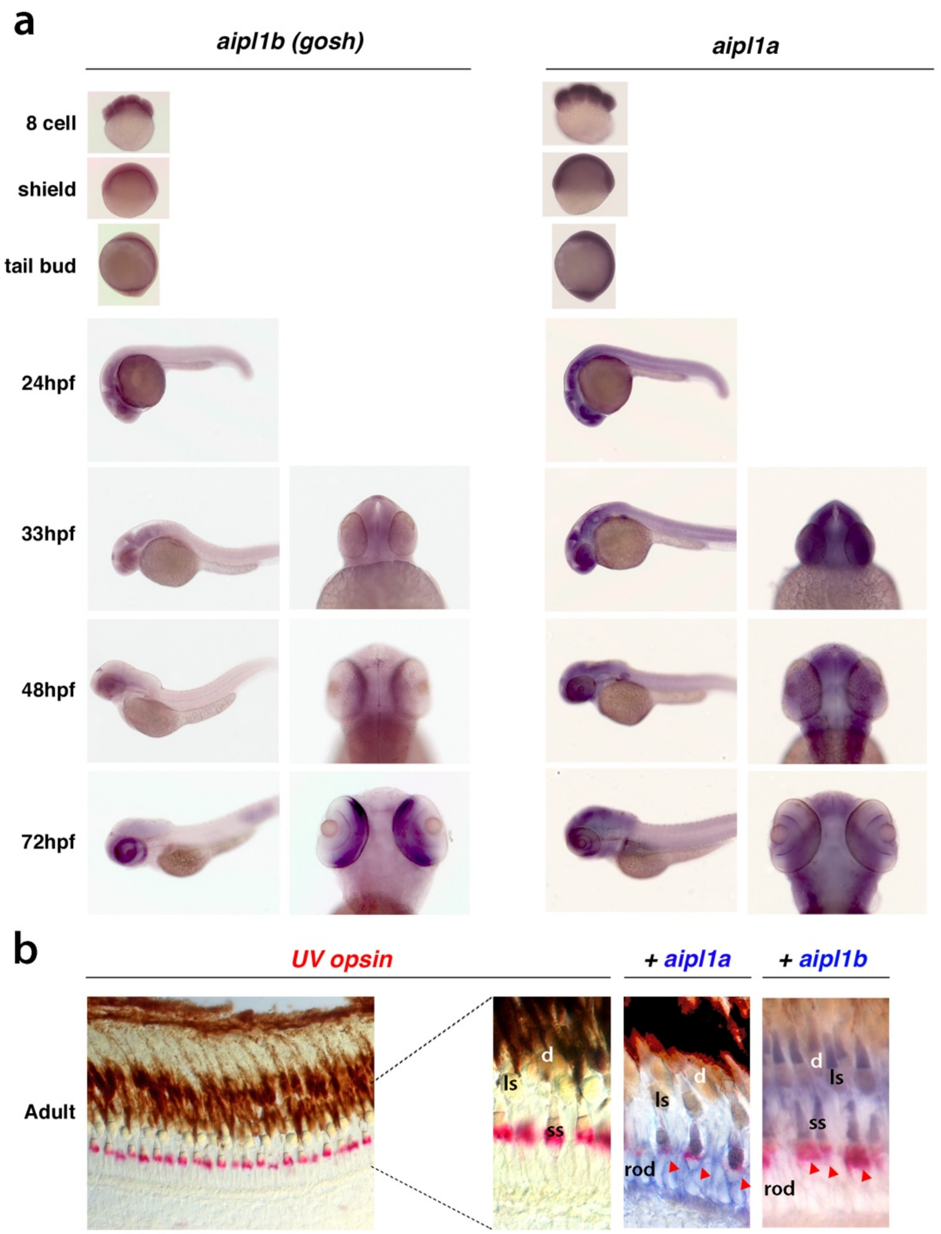

Figure S3. Expression of *aipl1* mRNA during embryonic development

(a) Whole-mount *in situ* hybridization of wild-type embryos from the 8-cell stage to 72 hpf using *aipl1b* and *aipl1a* RNA probes.

(b) (Left panel) *In situ* hybridization of cryo-sectioned wild-type adult retina with *UV opsin* RNA probe (red). UV opsin is expressed in short single cones, which are regularly located just above the nuclear layer of rod photoreceptors. (Right panels) Wild-type adult retinas were co-labeled with the *UV opsin* RNA probe (red), and the *aipl1a* or *aipl1b* probe (blue). *aipl1a* and *aipl1b* mRNAs are expressed in rods and cones, respectively. Only short single cones are positive for both *aipl1a* and *aipl1b* probes (red arrowhead). Abbreviation: ss, single short cone; ls, long single cone; d, double cone.

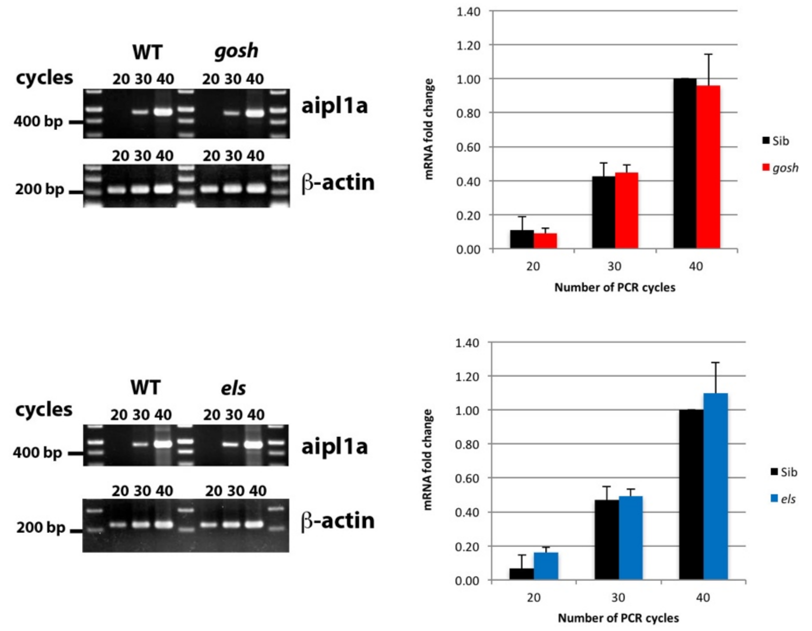

Figure S4. *Aipl1a* expression in *gosh* and *els* mutants is similar to that of wild type animals
Semi-quantitative PCR of *aipl1a* in wild type and *gosh* (upper panel) or *els* (lower panel) mutants at 7 dpf. PCR amplification at 20, 30, or 40 cycles is shown in lanes of electrophoresis (left panels). *Aipl1a* mRNA level is similar between wild-type and *gosh* or *els* mutant samples. β-actin is used as a control.

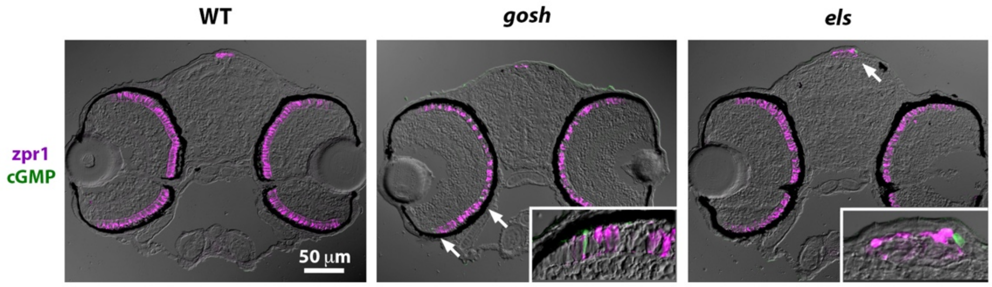

Figure S5. Labeling of wild-type, *gosh*, and *els* mutant retinas with anti-cGMP antibody
Cryo-sectioned heads of 4-dpf wild-type, *gosh*, and *els* mutant embryos are labeled with anti-formaldehyde-fixed cGMP antibody (green) and zpr1 antibody (magenta). As in 7-dpf samples (Fig. 5a), wild-type, *gosh*, and *els* mutant retinas show undetectable levels of cGMP, although a few photoreceptors with high levels of cGMP were occasionally observed in the CMZ of *gosh* and *els* mutant retinas (arrows in *gosh* mutant panel). High levels of cGMP were observed in pineal photoreceptors in both mutants (arrow in *els* mutant panel).
